# Gene expression and metabolic variability in fungus‐resistant PIWI (‘Pilzwiderstandsfähig’) grape cultivars

**DOI:** 10.1002/jsfa.70645

**Published:** 2026-04-12

**Authors:** Clizia Villano, Alessandra Luciano, Domenico Carputo, Luigi Frusciante, Martino Forino, Felice Napolitano, Roberta Smimmo, Angelita Gambuti, Riccardo Aversano

**Affiliations:** ^1^ Department of Agricultural Sciences University of Naples Federico II Avellino Italy

**Keywords:** glycosylation, polyphenols, qRT‐PCR, *Vitis vinifera*

## Abstract

**BACKGROUND:**

Fungus‐resistant PIWI (*Pilzwiderstandsfähig*) grape cultivars are promising for reducing pesticide inputs in viticulture, but their enological potential is still poorly characterized. We performed a pilot, single‐vintage study integrating berry gene expression and phenolic composition in five Italian red PIWI cultivars (Cabernet Eidos, Cabernet Volos, Julius, Merlot Kanthus and Merlot Khorus), using Pinot Noir as a non‐PIWI *Vitis vinifera* benchmark.

**RESULTS:**

At harvest, transcript levels of 15 genes involved in sugar/energy signalling (*TOR3, SnRK1.1, SnRK1.2*), flavonoid biosynthesis (*LAR1, LAR2, ANR, DFR, LDOX, FLS4, FLS5*) and flavonoid glycosylation (*GAT1, UFGT, Va5GT, Vl3GT, UGT72*), were quantified, together with technological maturity traits, skin and seed phenolic fractions, and skin flavonol profiles. The PIWI panel showed marked variety‐specific transcriptional and metabolic differences. SnRK1 isoforms varied more strongly among cultivars than TOR3. Hierarchical clustering of scaled transcript profiles separated samples into two major branches, indicating coordinated genotype‐dependent modulation of sugar‐signalling and flavonoid/glycosylation genes. Higher expression of flavonoid biosynthetic and glycosyltransferase genes (including LAR2, FLS5, Va5GT and UGT72) was associated with higher anthocyanins, quercetin‐ and myricetin‐type flavonol glycosides, and colour intensity. Several, but not all, PIWI cultivars combined moderate‐to‐high sugars with comparatively high acidity, frequent enrichment in skin anthocyanins and flavonol glycosides, and lower extractable skin tannins than Pinot Noir.

**CONCLUSION:**

These results support a cultivar‐resolved molecular and compositional framework for interpreting enological variability in Italian red PIWI grapes. The data may guide breeding and cultivar‐tailored winemaking strategies, while broader generalization requires validation across additional genotypes, vintages and ripening stages. © 2026 The Author(s). *Journal of the Science of Food and Agriculture* published by John Wiley & Sons Ltd on behalf of Society of Chemical Industry.

## INTRODUCTION

Grapevine (*Vitis vinifera* L. subsp. *vinifera*) is highly susceptible to fungal diseases like *Plasmopara viticola* (downy mildew) and *Erysiphe necator* (powdery mildew). Traditionally, these diseases have been effectively controlled through the intensive use of chemical pesticides, and in cool and wet climates up to 12 treatments per season may be required.^1^ While these types of applications remain very successful, they raise serious concerns regarding human health, environmental sustainability, and soil quality. Consequently, the European Union has made the reduction of pesticide use a policy priority, promoting the development and adoption of disease‐resistant grapevine cultivars.^2^ Over the last decades, extensive breeding programmes have focused on production of hybrids integrating resistance traits from wild American and Asian grapevine species into elite European *V. vinifera* cultivars. These efforts have culminated in the production of over 100 PIWI cultivars (from the German *pilzwiderstandsfähig*, meaning ‘fungus‐resistant’). Among the cultivated resistant cultivars, Bianca is one of the most widespread and covers 0.22% of the worldwide area cultivated with vines, mostly spread in Russia, Hungary, and Moldova. Other resistant cultivars comprise Villard Noir, Seyval Blanc, Chambourcin, and Regent.^3^, ^4^, ^5^, ^6^ The Italian contributions to PIWI breeding has been significant and led to the production of Cabernet‐ and Merlot‐derived cultivars such as ‘Cabernet Volos’ and ‘Merlot Kanthus’, developed through backcrossing strategies at the University of Udine (Italy) and the Istituto di Genomica Applicata (IGA, Udine, Italy).^7^ These cultivars were obtained by introgressing resistance loci (*Rpv*, *Ren* and *Run*) from wild grapevine species, followed by successive selections aimed at recovering high enological quality. These cultivars retain over 85% of the *V. vinifera* genome and have been approved for both conventional and organic farming in Europe, including the production of wines with protected designations of origin (PDO).^2^

Despite their promise, PIWI cultivars remain under‐characterized from a genetic and oenological perspective. A recent transcriptomic study confirmed distinct expression profiles in PIWI berries compared to their *V. vinifera* progenitors, including differential expressed genes related to organic acid metabolism and transport.^8^ Foria *et al*.^7^ showed that PIWI cultivars retain wild‐derived haplotype blocks (e.g., from *Vitis aestivalis*, *Vitis amurensis*) spanning 7–11 Mb around R loci, signifying linkage drag that may affect broader regulatory networks. These introgressed regions could influence hormone signalling and solute transport, potentially altering nutrients and metabolite movement during berry development and ripening. Several studies provided evidence that PIWI grapes often exhibit high levels of titratable acidity, low levels of condensed tannins, and high yeast‐assimilable nitrogen, which may ultimately impact fermentation dynamics and the sensory profile of wine.^9^, ^10^, ^11^ Yet, we have a limited and incomplete understanding of the enological behaviour of the Italian PIWI grapes with regard to fermentation and ageing, and scant knowledge of the metabolic composition of the berries, especially regarding skin and seed phenolics and glycosylated flavonoids, which are needed to predict their behaviour in the cellar. Without this knowledge, it is difficult to adapt vinification protocols or genetically choose the best clones to produce wines of reliable quality.

This study aimed to address these gaps through a pilot, single‐vintage investigation of the molecular and metabolic bases underlying the enological potential of selected Italian red PIWI grape cultivars, using Pinot Noir as a non‐PIWI *V. vinifera* benchmark. We hypothesized that cultivar‐dependent differences in berry transcriptional and phenolic profiles may be influenced by both resistance‐related introgressions and the broader genetic background resulting from breeding history. In this context, Pinot Noir was selected as a well‐characterized red *V. vinifera* non‐PIWI reference to provide a single common benchmark for transcriptional and phenolic comparisons across this small and genetically heterogeneous PIWI panel. This choice was intended to anchor all comparisons within the same analytical framework, rather than to test phenolic or genetic equivalence with the *V. vinifera* progenitors of each PIWI cultivar. To test this working hypothesis, we quantified the absolute transcript abundance of key genes involved in sugar and energy signalling, flavonoid biosynthesis, and flavonol glycosylation, and we measured technological maturity traits, polyphenolic compounds extracted from grape skins, and seed flavonol profiles by high‐performance liquid chromatography (HPLC). By integrating transcriptional and chemical data, this work provides a first cultivar‐resolved framework to support future breeding‐ and winemaking‐oriented investigations.

## MATERIAL AND METHODS

### Plant materials

The grapevine germplasm analysed consisted of *V. vinifera* cv. Pinot Noir (PN), used as a non‐PIWI *V. vinifera* reference cultivar, and five PIWI grapevine cultivars: Merlot Kanthus (KNT), Merlot Khorus (KHR), Cabernet Eidos (EDS), Cabernet Volos (VLS), and Julius (JLS). Detailed information on cultivar identity, reported pedigree/cross, resistant donor parent, and reported resistance loci/QTL is provided in Supporting Information, Table S1. The vines used in the experiment were grown in an experimental vineyard located in Pietradefusi (province of Avellino, Campania, southern Italy), and were all grafted onto SO4 rootstock, spaced 2.5 m × 1.0 m (corresponding to 4000 vines ha^−1^), and trained to a unilateral Guyot system with an average of ten buds per vine (eight buds on the long cane and two buds on the spur). Vineyard management followed standard local practices for the area. In 2023, grapes from each cultivar were sampled at harvest on cultivar‐specific dates, when each genotype had reached its own technological maturity for red wine production. For each cultivar, approximately 200 berries were randomly collected and used to obtain three independent biological replicates. Each biological replicate consisted of a pooled sample of berries originating from five clusters harvested from five different vines. After sampling, the berries were immediately stored at −80 °C, and the same biological replicates were subsequently used for all chemical and gene expression analyses.

### Transcriptional analyses

RNA was extracted following the protocol described by Japelaghi *et al*.,^12^ using 1.0 g of ground berries after removing seeds and pedicels. The concentration and purity of the isolated RNA were assessed using a NanoDrop ND‐1000 spectrophotometer (Thermo Fisher Scientific, Wilmington, DE, USA). RNA integrity was confirmed via 0.8 g L^−1^ % (agarose gel electrophoresis, visualized with the Gel Doc XR system (Bio‐Rad Laboratories Inc., Munich, Germany)). For complementary DNA (cDNA) synthesis, 500 ng of each RNA sample was reverse‐transcribed using the RevertAid First Strand cDNA Synthesis Kit (Thermo Fisher Scientific) according to the manufacturer's instructions. The synthesized cDNA was diluted 1:5 prior to quantitative real‐time polymerase chain reaction (qRT‐PCR) analysis. Fifteen genes were selected for absolute gene expression analysis. They were chosen based on their roles in metabolic pathways of enological interest, including sugar synthesis, flavonoid and phenolic biosynthesis, and flavonoid glycosylation (Table S2). In particular, TOR3, SnRK1.1, and SnRK1.2 are involved in energy and sugar metabolism,^13^, ^14^, ^15^ influencing carbon allocation and the balance between anabolic and catabolic processes; LAR1, LAR2, GAT1, and ANR are directly associated with the biosynthesis of polyphenolic compounds,^16^, ^17^, ^18^ particularly the flavan‐3‐ol/proanthocyanidin branches of flavonoid pathway; DFR, LDOX, FLS4 and FLS5 participate in the core steps of flavonoid biosynthetic pathway, contributing to the production of anthocyanins and flavonols^19^, ^20^, ^21^; UFGT, 5GT4, 3GT4, and UGT72 encode glycosyltransferases that catalyse flavonoid glycosylation, affecting their stability, solubility and bioactivity.^22^, ^23^, ^24^ Absolute quantification of gene expression was performed using qRT‐PCR. Standard curves for each target gene were generated from serial dilutions of purified PCR amplicons, enabling absolute quantification of messenger RNA (mRNA) copy number in each sample.^25^, ^26^ The qRT‐PCR analysis was conducted in a 10 μL volume containing 2× SYBR Green Master Mix (Thermo Fisher Scientific), 10 μmol L^−1^ of each forward and reverse primer, and 2 μL of 1:5 diluted cDNA. Each reaction was performed in triplicate using the QuantStudio™ 3 Real‐Time PCR System (Thermo Fisher Scientific). The overall qRT‐PCR set‐up and validation followed previously established procedures for flavonoid‐pathway genes.^27^

### Metabolic analysis

Separate extraction of berry components was carried out in duplicate simulating the maceration process necessary to produce red wines.^28^ Standard chemical analyses and spectrophotometric measurements were carried out to assess grape composition, including soluble solids, titratable acidity and pH, according to the OIV Compendium of International Methods of Analysis of Wine and Musts.^29^ Flavanols were determined as vanillin reactive flavans,^30^ while total phenolics, BSA‐reactive tannins and total anthocyanins were determined by the Harbertson–Adams assay.^31^

For the separation, identification, and quantification of flavonols, HPLC was performed using an Agilent 1260 Infinity II LC system (Agilent Technologies, Santa Clara, CA, USA) equipped with a binary pump (G7112B), an integrated dual‐channel degasser unit and an ultraviolet (UV)‐visible detector (G7114A). Data acquisition and processing were performed using OpenLAB CDS ChemStation Edition software (Agilent Technologies). All solvents were of HPLC quality and all chemicals of analytical grade (> 99%). Water (ultrapure), acetonitrile (99.9%), methanol (99.9%) and acetic acid (≥ 99.5%) were from Sigma‐Aldrich (Milan, Italy). Commercial standard of quercetin dihydrate (> 99%), was obtained from Extrasynthese (Genay, France). Sample filtration was carried out using 0.45 μm membrane filters (Chromafil, Germany). Chromatographic separation was carried out using a Poroshell 120 EC‐C18 column (250 mm × 4.6 mm. 2.7 μm) in combination with an ultrahigh‐performance liquid chromatography (UHPLC) guard pre‐column (4.6 mm × 5 mm. 2.7 μm). A volume of 10 μL of calibration standard or sample extracts was injected into the column. All samples were filtered through 0.45 μm Durapore membrane filters (Millipore, Carrigtohill, Ireland), transferred into glass vials, and immediately analysed using the HPLC system. The mobile phase was set at a flow rate of 0.500 mL min^−1^ and consisted of (solvent A) 0.3% acetic acid in ultrapure water–acetonitrile mixture (8:1 *v/v*); and (solvent B) 0.3% acetic acid in ultrapure water–acetonitrile (4:5 *v/v*). The following gradient was used: 0–45 min from 0 to 35% B, 45–50 min from 35 to 100% B, 50–55 min from 100 to 100% B, 55–58 min from 100 to 0% B, 58–64 min from 0 to 0% B. The detection wavelength was set at 360 nm.

### Statistical analysis of transcriptional and metabolic data and correlation tests

For each cultivar, three biological replicates were used as experimental units. All measurements were carried out in triplicate (technical replicates) on each biological replicate, and the mean of the three technical replicates was used for statistical analysis. The statistical analysis of absolute gene expression values obtained from standard curves for each gene across the different cultivars was performed using a one‐way analysis of variance (ANOVA), followed by Tukey's honestly significant difference (HSD) test for *post hoc* pairwise comparisons. ANOVA was run only when the data showed normality and homogeneity of variances; otherwise, a non‐parametric Kruskal–Wallis test was applied. When the Kruskal–Wallis test was significant (*P* < 0.05), the Bonferroni–Dunn test was used for pairwise comparisons. For the heatmap, mean gene expression values were centred and scaled per gene (*z*‐score; mean = 0, standard deviation = 1 across cultivars) and plotted with hierarchical clustering. The colour scale therefore reflects relative expression for each gene, not absolute values or fold changes. Metabolic quantitative data were analysed using the same approach. Relationships between gene expression and metabolic parameters were investigated using principal component analysis (PCA) and Pearson correlation coefficients (*P* < 0.05). All values were expressed as means ± standard error of three biological replicates. All statistical analyses were performed using XLSTAT software (version 2013.6.04; Addinsoft, Paris, France).

## RESULTS

### Genotype‐dependent expression of sugar, flavonoid and glycosylation genes

**Figure 1:**
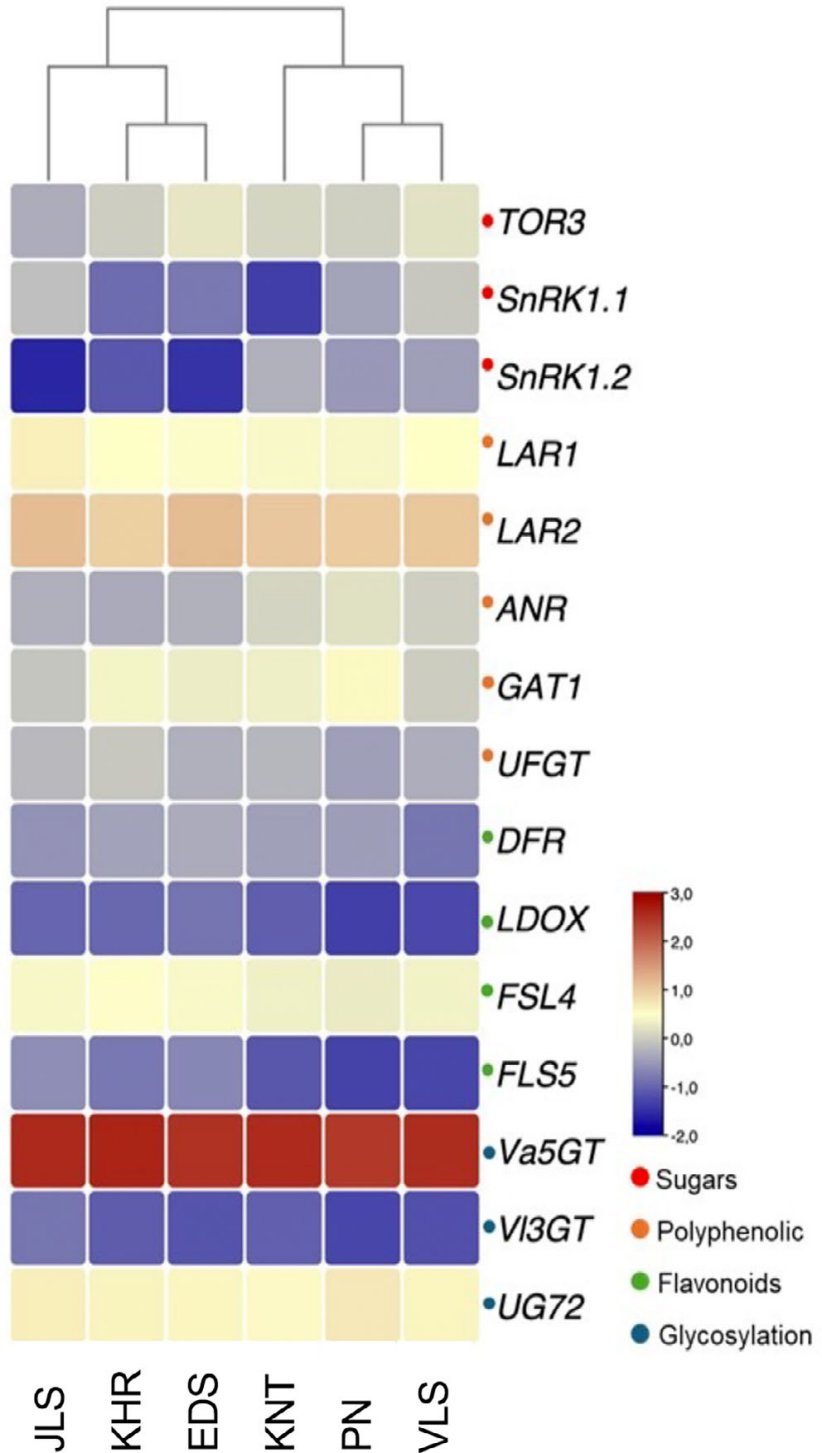
Heatmap representing scaled expression values (*z‐*scores) of 15 target genes in berries of Pinot Noir (PN) and PIWI cultivars. Rows correspond to genes involved in sugar signalling (red dot), polyphenol biosynthesis (orange dot), flavonoids (green dot) and glycosylation (blue dot). Columns correspond to cultivars Julius (JLS), Merlot Khorus (KHR), Cabernet Eidos (EDS), Merlot Kanthus (KNT), Pinot Noir (PN), Cabernet Volos (VLS). Values are *z*‐scores and colours indicate relative expression (red = above, blue = below the average for that gene).

The expression profiles of 15 target genes were analysed in all five PIWI cultivars and compared with PN as a *V. vinifera* benchmark (Fig. 1 and Supporting Information, Table S3). Among sugar‐signalling genes, TOR3 varied within a narrow range, with VLS having the highest and JLS the lowest transcript levels, while EDS, KNT and KHR were close to PN. By contrast, SnRK1 isoforms showed broader modulation: SnRK1.1 was highest in VLS and JLS, intermediate in PN and reduced in EDS, KHR and especially KNT, whereas SnRK1.2 was high in PN, VLS and KNT, intermediate in KHR and lowest in JLS and EDS. For core flavonoid biosynthesis, LAR2 consistently exceeded LAR1 in all cultivars, ANR varied only moderately (with PN and VLS at the upper end), and DFR was highest in PN with slightly lower, overlapping values in the PIWI genotypes. LDOX transcripts were marginally higher in EDS than in PN and in a similar range in the remaining cultivars. Late flavonoid and glycosyltransferase genes showed clearer expression ranking. FLS4 accumulated to higher levels than both FLS5 and LDOX across all cultivars, while FLS5 varied more strongly among genotypes, with JLS and EDS at the upper end and PN and KNT at the lower end (Fig. 1). In the glycosylation pathway, Va5GT was the most abundant transcript, followed by UGT72, whereas Vl3GT had the lowest expression in every cultivar. Within this ranking, JLS and KHR tended to have the highest Va5GT levels and PN the lowest, while GAT1 and UFGT varied within a narrower range, with PN and KHR slightly above the other genotypes (Table S3).

Hierarchical clustering of the scaled expression data across all 15 genes (Fig. 1) separated the cultivars into two main groups: JLS–EDS–KHR on one branch and PN–KNT–VLS on the other. This pattern summarizes the combined effects of the individual gene differences described earlier, indicating that PN is transcriptionally more similar to KNT and VLS than to JLS, EDS and KHR, and that a subset of PIWI cultivars share a coordinated shift in sugar‐signalling and flavonoid/glycosylation genes. To relate these global transcriptional patterns to berry composition, we next examined the phenolic profiles of skins and seeds.

### Basic metabolic and phenolic composition of PIWI and PN berries

With regard to basic compositional traits, KNT and especially EDS had higher soluble solids than the other cultivars, while their pH and titratable acidity remained within the usual range for red grapes. In contrast, VLS, JLS and particularly KHR combined lower pH with higher titratable acidity than PN and KNT (Table 1). Skin and seed phenolics, quantified as iron‐reactive phenolics, vanillin‐reactive flavans, BSA‐reactive tannins and, in skins, total anthocyanins, also differed among cultivars (Tables 2 and 3). In particular, in berry skins, EDS had the highest levels of total phenolics, flavanols and tannins, whereas JLS showed the highest total anthocyanin content. PN and KNT had the lowest anthocyanin concentrations, with VLS and KHR at intermediate levels. In seeds, PN showed the highest total phenolic content, while EDS had the highest flavanol and tannin levels. Across all cultivars, seeds contained much higher amounts of flavans and BSA‐reactive tannins than skins, whereas in all PIWI cultivars total iron‐reactive phenolics were higher in skins than in seeds. Consistent with these patterns, colour intensity and absorbance at 420, 520 and 620 nm of hydroalcoholic extracts obtained from skins were higher in all PIWI cultivars than in PN, with JLS and VLS showing the most intense colour, KNT and KHR intermediate values, and EDS the lowest among PIWIs; hue values indicated relatively redder tones in VLS/JLS and more yellowish hues in KNT (Table 4).

**Table 1:** Basic berry composition of Pinot Noir (PN) and PIWI cultivars at harvest

Sample	Berry weight (g)	Soluble solids (°Brix)	pH	Titratable acidity (g L^−1^ tartaric acid)
PN	1.10 ± 0.02BC	24.00 ± 0.00C	3.37 ± 0.01CD	8.40 ± 0.00D
JLS	1.40 ± 0.04AB	23.65 ± 0.06D	3.28 ± 0.01CD	8.68 ± 0.05C
EDS	0.94 ± 0.04C	28.48 ± 0.05A	3.41 ± 0.00B	7.75 ± 0.06E
VLS	1.34 ± 0.00AB	23.70 ± 0.00D	3.29 ± 0.01C	8.90 ± 0.00B
KNT	1.58 ± 0.12A	26.70 ± 0.00B	3.59 ± 0.02A	6.63 ± 0.05F
KHR	1.20 ± 0.02B	23.70 ± 0.00D	3.26 ± 0.01D	9.53 ± 0.05A

*Note*: Reported parameters are berry weight, soluble solids (°Brix), must pH and titratable acidity (g L^−1^, expressed as tartaric acid equivalents). Values are mean ± standard error. Different uppercase letters within a column indicate significant differences among grape varieties (*P* < 0.05). Cultivar abbreviations are as follows: PN, Pinot Noir; VLS, Cabernet Volos; JLS, Julius; EDS, Cabernet Eidos; KNT, Merlot Kanthus; KHR, Merlot Khorus.

**Table 2:** Phenolic composition of skin extracts obtained by simulated maceration of Pinot Noir (PN) and PIWI cultivars: total phenolics, flavans, tannins and total anthocyanins (mg g^−1^ fresh weight (FW))

Sample	Total phenolics (mg g^−1^ FW)	Flavans (mg g^−1^ FW)	Tannins (mg g^−1^ FW)	Total anthocyanins (mg g^−1^ FW)
PN	9.200 ± 0.909AB	2.916 ± 0.416AB	2.645 ± 0.202B	3.391 ± 0.219C
JLS	11.006 ± 1.095AB	2.479 ± 0.336AB	1.912 ± 0.488B	9.575 ± 0.723A
EDS	16.125 ± 6.971A	4.790 ± 2.507A	6.990 ± 0.952A	6.649 ± 2.214B
VLS	10.999 ± 2.700AB	3.990 ± 1.089AB	1.798 ± 0.455B	6.456 ± 0.292B
KNT	7.832 ± 0.406B	1.908 ± 0.063B	1.769 ± 0.565B	3.131 ± 0.168C
KHR	9.344 ± 0.809AB	2.383 ± 0.152AB	1.516 ± 0.378B	5.174 ± 0.678 BC

*Note*: Values are mean ± standard error. Different uppercase letters within a column indicate significant differences among cultivars (*P* < 0.005). Cultivar abbreviations are as follows: PN, Pinot Noir; VLS, Cabernet Volos; JLS, Julius; EDS, Cabernet Eidos; KNT, Merlot Kanthus; KHR, Merlot Khorus.

**Table 3:** Seed phenolic composition extracted by simulated maceration in Pinot Noir (PN) and PIWI cultivars: total phenolics, flavans and tannins (mg g^−1^ fresh weight (FW))

Sample	Total phenolics (mg g^−1^ FW)	Flavans (mg g^−1^FW)	Tannins (mg g^−1^ FW)
PN	11.914 ± 0.643A	26.125 ± 3.640BC	15.700 ± 1.220B
JLS	8.733 ± 0.370B	26.815 ± 1.423BC	8.747 ± 0.480DE
EDS	8.030 ± 0.566B	71.111 ± 8.461A	24.579 ± 0.465A
VLS	6.300 ± 0.470C	33.745 ± 4.185B	9.678 ± 0.517D
KNT	4.736 ± 0.899D	13.753 ± 2.551D	7.032 ± 0.897E
KHR	2.834 ± 0.745E	20.874 ± 1.683CD	12.394 ± 1.062C

*Note*: Values are mean ± standard error. Different uppercase letters within a column indicate significant differences among cultivars (*P* < 0.005). Cultivar abbreviations are as follows: PN, Pinot Noir; VLS, Cabernet Volos; JLS, Julius; EDS, Cabernet Eidos; KNT, Merlot Kanthus; KHR, Merlot Khorus.

**Table 4:** Chromatic parameters of skin extracts from Pinot Noir (PN) and PIWI cultivars: colour intensity, hue and absorbance at 420, 520 and 620 nm

Sample	Colour intensity	Hue	420 nm	520 nm	620 nm
PN	4.25 ± 0.08E	0.60 ± 0.01B	1.49 ± 0.02E	2.49 ± 0.06E	0.28 ± 0.01E
JLS	11.09 ± 0.53A	0.49 ± 0.00D	3.37 ± 0.17A	6.85 ± 0.33A	0.87 ± 0.05A
EDS	5.36 ± 0.11D	0.60 ± 0.01B	1.89 ± 0.05D	3.14 ± 0.04D	0.34 ± 0.03E
VLS	9.91 ± 0.35B	0.50 ± 0.00D	3.03 ± 0.10B	6.10 ± 0.23B	0.78 ± 0.03B
KNT	7.65 ± 0.36C	0.73 ± 0.02A	2.94 ± 0.09B	4.04 ± 0.24C	0.68 ± 0.04C
KHR	7.28 ± 0.27C	0.54 ± 0.01C	2.40 ± 0.09C	4.43 ± 0.16C	0.45 ± 0.03D

*Note*: Values are mean ± standard error. Different uppercase letters within a column indicate significant differences among cultivars (*P* < 0.005). Cultivar abbreviations are as follows: PN, Pinot Noir; VLS, Cabernet Volos; JLS, Julius; EDS, Cabernet Eidos; KNT, Merlot Kanthus; KHR, Merlot Khorus.

Flavonol analysis of skin extracts confirmed that these compounds occurred predominantly as quercetin‐ and myricetin‐type glycosides (Table 5). The sum of quercetin glycosides ranged from about 480 to 1709 mg kg^−1^ fresh weight (FW), with JLS and KHR at the upper end and PN, KNT and EDS at the lower end. Myricetin‐3‐glucoside was highest in JLS, followed by PN and VLS, and lowest in KNT, EDS and KHR. Free quercetin was detected only in PN and VLS, while free myricetin occurred at low levels in VLS and JLS. The marked differences in berry composition among cultivars raised the question of how they relate to the underlying transcriptional dynamics. We therefore integrated gene expression and metabolic data using multivariate and correlation analyses.

**Table 5:** Flavonol composition of skin extracts from Pinot Noir (PN) and PIWI cultivars: quercetin, myricetin, sum of quercetin glycosides and myricetin‐3‐glucoside (mg kg^−1^ fresh weight (FW))

Sample	Quercetin (mg kg^−1^ FW)	Myricetin (mg kg^−1^ FW)	Sum of quercetin glycosides (mg kg^−1^ FW)	Myricetin‐3‐glucoside (mg kg^−1^ FW)
PN	2.82 ± 0.71B	n.d.	723.46 ± 7.94D	233.29 ± 5.38B
JLS	n.d.	8.35 ± 2.28A	1708.60 ± 11.35A	336.87 ± 40.79A
EDS	n.d.	n.d.	481.60 ± 0.27E	85.28 ± 20.86C
VLS	5.39 ± 0.88A	7.38 ± 0.52A	1044.12 ± 53.50C	204.52 ± 3.03B
KNT	n.d.	n.d.	531.76 ± 24.43E	40.20 ± 5.18D
KHR	n.d.	n.d.	1382.49 ± 117.34B	82.74 ± 5.30CD

*Note*: Values are mean ± standard error. Different uppercase letters within a column indicate significant differences among cultivars (*P* < 0.005); n.d., not detected. Cultivar abbreviations are as follows: PN, Pinot Noir; VLS, Cabernet Volos; JLS, Julius; EDS, Cabernet Eidos; KNT, Merlot Kanthus; KHR, Merlot Khorus.

### Correlation between gene expression and phenotypic traits

Pearson correlation analysis identified multiple significant associations between gene expression and phenotypic variables (*P* < 0.05), highlighting potential regulatory links between gene expression and berry composition (Table 6). In the sugar metabolism pathway, TOR3 expression showed a strong negative correlation with myricetin content (*r* = −0.960). For polyphenol biosynthesis genes, LAR1 expression was positively correlated with skin anthocyanins (*r* = 0.805), colour intensity (*r =* 0.853), absorbance at 520 nm (*r* = 0.912), and the sum of quercetin glycosides (*r* = 0.904). Similarly, LAR2 also showed strong correlation with skin anthocyanins (*r* = 0.979), colour intensity (*r* = 0.786), and 520 nm absorbance (*r* = 0.841). Within the flavonoid pathway, FLS4 showed strong negative correlations with pH (*r* = −0.876) and hue (*r* = −0.887), while FLS5 expression was positively associated with skin anthocyanins (*r* = 0.921). Regarding glycosylation, Va5GT expression showed high correlations with quercetin glycosides (*r* = 0.863). Vl3GT was strongly correlated with colour parameters at 420, 520, and 620 nm (*r* = 0.733, *r* = 0.794, and *r* = 0.843, respectively), and UGT72 expression was associated with myricetin‐3‐glucoside levels (*r* = 0.891).

**Table 6:** Pearson correlation coefficients between berry composition traits and gene expression levels in Pinot Noir (PN) and PIWI cultivars

Variable	TOR3	SnRK1.1	SnRK1.2	LAR1	LAR2	ANR	GAT1	DFR	LDOX	FLS4	FLS5	Va5GT	Vl3GT4	UFGT	UGT72
Berry weight (g)	−0.267	−0.034	0.389	0.316	0.123	0.282	−0.389	−0.530	−0.111	0.072	−0.204	0.218	0.657	0.455	0.081
Soluble solids (°Brix)	0.198	−0.626	−0.186	−0.686	−0.278	−0.308	−0.055	0.332	0.368	−0.670	−0.003	−0.372	−0.612	−0.724	**−0.878**
pH	0.083	−0.642	0.378	−0.708	−0.568	0.211	0.135	0.358	−0.147	**−0.876**	−0.500	−0.592	−0.248	−0.604	−0.811
Titratable acidity (g L^−1^ TA)	−0.032	0.522	−0.262	0.593	0.375	−0.176	0.056	−0.276	−0.014	**0.853**	0.324	0.546	0.144	0.661	0.745
Skin total phenolics (mg g^−1^ FW)	0.229	0.100	−0.592	−0.037	0.385	−0.460	−0.342	−0.017	0.752	0.034	0.574	0.052	−0.353	−0.387	−0.157
Skin flavans (mg g^−1^ FW)	0.638	0.283	−0.173	−0.126	0.195	−0.061	−0.122	−0.037	0.708	0.007	0.207	−0.229	−0.307	−0.348	−0.091
Skin tannins (mg g^−1^ FW)	0.244	−0.210	−0.442	−0.453	−0.019	−0.372	−0.028	0.380	0.479	−0.394	0.296	−0.290	−0.617	−0.727	−0.491
Skin total anthocyanins (mg g^−1^ FW)	−0.356	0.522	−0.698	**0.805**	**0.979**	−0.455	**−0.830**	−0.613	0.646	0.658	**0.921**	0.717	0.453	0.332	0.546
Seeds total phenolics (mg g^−1^ FW)	−0.064	0.528	0.017	0.025	0.095	0.415	0.118	0.435	−0.154	−0.341	0.094	−0.487	0.198	−0.599	0.318
Seeds flavans (mg g^−1^ FW)	0.352	0.038	−0.466	−0.210	0.208	−0.358	−0.182	0.121	0.677	−0.114	0.411	−0.132	−0.462	−0.510	−0.265
Seeds tannins (mg g^−1^ FW)	0.277	−0.156	−0.414	−0.478	−0.159	−0.343	0.270	0.550	0.248	−0.294	0.207	−0.310	−0.737	−0.654	−0.354
Colour intensity	−0.269	0.435	−0.129	**0.853**	0.786	−0.070	**−0.828**	**−0.934**	0.436	0.679	0.440	0.706	0.798	0.729	0.530
Hue	0.067	−0.764	0.384	**−0.827**	−0.753	0.162	0.374	0.532	−0.347	**−0.887**	−0.596	−0.613	−0.422	−0.591	**−0.850**
Absorbance at 420 nm	−0.286	0.212	−0.043	0.688	0.636	−0.066	−0.806	**−0.885**	0.381	0.500	0.310	0.627	0.733	0.655	0.308
Absorbance at 520 nm	−0.258	0.525	−0.186	**0.912**	**0.841**	−0.092	**−0.814**	**−0.931**	0.454	0.761	0.503	0.741	0.794	0.754	0.625
Absorbance at 620 nm	−0.235	0.374	0.039	0.734	0.675	0.088	**−0.812**	**−0.883**	0.384	0.471	0.285	0.529	**0.843**	0.600	0.409
Quercetin (mg kg^−1^ FW)	0.427	0.234	0.076	0.406	0.470	−0.047	−0.613	**−0.897**	0.765	0.540	0.132	0.402	0.315	0.577	0.107
Myricetin (mg kg^−1^ FW)	**−0.960**	−0.116	−0.676	0.294	0.363	−0.498	−0.308	0.174	−0.222	0.002	0.643	0.426	0.159	−0.116	0.145
Sum quercetin glycosides (mg kg^−1^ FW)	−0.569	0.462	−0.430	**0.904**	0.704	−0.303	−0.436	−0.552	0.009	**0.871**	0.600	**0.863**	0.570	0.786	0.798
Myricetin‐3‐glucoside (mg kg^−1^ FW)	−0.354	**0.874**	−0.154	0.780	0.677	0.254	−0.365	−0.269	0.010	0.419	0.455	0.242	0.758	0.199	**0.891**

*Note*: Values in bold are significantly different from zero (*P* < 0.05). Strong correlations (|*r|* > 0.80) are highlighted (red for positive, black for negative). FW, fresh weight; TA, tartaric acid equivalents.

## DISCUSSION

The composition of sugars, polyphenols, and their glycosylated derivatives in grape berries has been widely studied across *V. vinifera* cultivars, revealing key insights into the molecular mechanisms driving fruit and wine quality.^32^, ^33^ In contrast, limited information is available for PIWI grapes, particularly for Italian red cultivars. The present study addresses this gap by integrating transcriptional and metabolic profiling of five Italian red PIWI cultivars and a *V. vinifera* benchmark (PN), with a specific focus on genes related to sugar metabolism, polyphenol biosynthesis, and flavonoid glycosylation, alongside phenolic characterization of berry skins and seeds. Given the limited panel and single harvest stage, the results are best interpreted as a cultivar‐resolved pilot framework.

### Modulation of sugar signalling and flavonoid‐associated gene expression in PIWI grape cultivars

Our gene expression data suggest that, in these cultivars, SnRK1 rather than TOR3 accounts for most of the variation within the TOR/SnRK1 pathway. TOR3 least‐squares means (LS means) varied within a relatively narrow range among genotypes and did not show a systematic PIWI *versus* PN trend, even though genotype effects were statistically significant. By contrast, SnRK1.1 and SnRK1.2 displayed broader, genotype‐dependent differences, with SnRK1.1 tending to be lower in EDS, KNT and KHR and SnRK1.2 reduced in JLS, EDS and KHR. In Arabidopsis, TOR and SnRK1 have been described as antagonistic regulators of energy status, with TOR promoting growth‐related (anabolic) processes and SnRK1 favouring energy‐conserving, catabolic responses under stress.^34^, ^35^, ^36^ Although we did not observe a simple reciprocal expression pattern for TOR3 and SnRK1 in berries at harvest, the genotype‐specific modulation of SnRK1 suggests that sugar‐ and energy‐status signalling is tuned differently among PIWI cultivars and PN, and may contribute to their distinct secondary metabolic profiles.

Consistent with the genotype‐dependent differences in sugar signalling described earlier, several flavonoid‐pathway genes tended to be expressed at slightly higher levels in some PIWI cultivars, particularly JLS, EDS and KHR, than in PN. LAR1 and LAR2 transcripts varied within a relatively narrow range across PN and the PIWI cultivars, yet both genes were positively correlated with total skin anthocyanins, colour intensity and absorbance at 520 nm, suggesting that even modest changes in their expression may contribute to variation in pigment accumulation. Among the late flavonoid genes, FLS5 showed the clearest genotype effect, with JLS exhibiting the highest transcript levels, in agreement with its stronger red colour and higher myricetin‐derived flavonols. Taken together, these associations are consistent with LAR2, LAR1 and FLS5 participating in the modulation of berry colour in these cultivars, in line with previous reports linking variation in LAR and FLS gene families to changes in grape skin flavonoid profiles.^37^, ^38^ More broadly, the co‐variation between SnRK1 transcripts and flavonoid‐related genes supports the idea that sugar‐ and energy‐status signalling, including the TOR/SnRK1 module, can influence flux through the phenylpropanoid and flavonoid pathways.^39^, ^40^ However, our single‐time‐point data do not allow us to resolve the underlying regulatory mechanisms in detail.

One of the most interesting findings was the strong activation of glycosylation‐related genes involved in a process that helps stabilize pigments and makes phenolic compounds more soluble. Among these, Va5GT showed the highest transcript abundance levels in all genotypes, with slightly higher expression levels in PIWI cultivars than in PN. Its expression was closely correlated with the sum of quercetin glycosides, suggesting that Va5GT plays an important role in adding sugars to flavonols. This is in line with observations in *V. amurensis*, where an anthocyanin 5‐O‐glucosyltransferase stabilizes red pigments by forming more stable diglucosides.^24^ Interestingly, Vl3GT displayed only moderate variation in expression among cultivars but was strongly associated with absorbance at 420, 520 and 620 nm, suggesting a contribution to the pool of colour‐related glycosides, similar to the roles reported for VvGT5 and VvGT6.^41^ UGT72 also varied moderately across genotypes and was linked to myricetin‐3‐glucoside levels, pointing to a more conserved contribution to flavonoid homeostasis. Taken together, these glycosyltransferases likely influence the stability, solubility and partitioning of polyphenols, properties that in turn affect wine traits such as colour retention, flavonol instability and aroma release over time.^42^ Although the correlations do not establish causality, their consistency across PIWI genotypes indicates that glycosylation pathways warrant further functional investigation in these cultivars.

### Cultivar‐specific patterns in acidity and phenolic composition across the PIWI grapes

The metabolic data of PIWI grapes revealed distinct profiles that both align with and diverge from those of *V. vinifera* cultivars, offering important insights into their enological potential. Interestingly, some of these phenotypic traits appear to be shaped by genotype‐specific transcriptional dynamics, as suggested by the correspondence between metabolic patterns and gene expression clustering. In this PIWI panel most cultivars exhibited high titratable acidity and moderate soluble solids, with KNT and especially EDS representing exceptions with higher sugar accumulation. Such values do not always reflect optimal technological maturity, and may introduce challenges in winemaking, especially concerning sensory balance. Similar features have been reported for other PIWI^11^, ^43^ and are likely influenced by wild *Vitis* ancestry, which often maintains high levels of malic acid due to limited post‐*veraison* degradation.^44^ Climatic adaptation may also contribute to the observed variability,^45^ as PIWI genotypes bred in colder northern environments may exhibit differential regulation of ripening‐related pathways under Mediterranean conditions.

Another recurrent feature observed in several of the analysed PIWI cultivars was the high level of total anthocyanins. This result is in agreement with the literature, as the phenolic profile of PIWI cultivars often differ from that of *V. vinifera* grapes with greater levels of several phenolic classes and a higher relative contribution of anthocyanins.^11^, ^46^ This is a typical characteristic of hybrid grapes. In a previous study, Ban *et al*.^46^ reported a wide, continuous variation in berry skin anthocyanic content among interspecific hybrid grapes (*Vitis labruscana × V. vinifera*), mainly attributable to genotypic effects. Variability among PIWI cultivars was substantial also for other metabolites. For instance, VLS and JLS showed elevated flavonol glycoside levels, potentially linked to higher expression of *Va5GT* and *Vl3GT*, while EDS displayed particularly high flavanol and tannin concentrations, in line with the expression of *LAR2* and anthocyanin pathway genes. KHR combined intermediate flavanol levels with relatively high anthocyanins and quercetin glycosides. The correspondence between these metabolic profiles and the transcriptional clustering of samples supports the view that metabolic divergence among the analysed cultivars is associated with genetic background and differential expression of genes involved in flavonoid biosynthesis and transport. In this context, integrating such transcriptional‐metabolic information with DNA‐based characterization of grapevine germplasm will be essential to fully resolve PIWI genomic backgrounds.^47^ PN grouped more closely with KNT (and to a lesser extent with VLS) in both metabolic and transcriptional analyses, suggesting partial conservation of *V. vinifera*‐like traits in these cultivars, whereas JLS, EDS and KHR demonstrated more divergent profiles.

Skins were generally less rich in extractable tannins compared to seeds, as observed also in other interspecific hybrids.^48^, ^49^ BSA‐reactive tannins from skin extracts were significantly lower in PIWI samples than in PN, with the exception of EDS. This may indicate that in PIWI berries even appreciable LAR2 expression does not translate into highly extractable condensed tannins in the skin, likely due to strong interactions with cell wall components. Previous studies have shown that hybrid grapes often exhibit stronger binding of tannins to cell‐wall materials, reducing their extractability during maceration.^50^, ^51^, ^52^ These interactions compromise tannin recovery and contribute to lower levels of polymeric pigments in the final wine, potentially limiting ageing potential and mouthfeel complexity.^53^, ^54^ The limited extractability of skin tannins contrasts with the higher abundance of seed tannins and flavanols observed in all PIWI cultivars. However, their contribution to wine structure remains uncertain, as these compounds may interact with polysaccharides and proteins from the skin and pulp, reducing their stability and functional integration.^55^ Furthermore, grape seed surfaces adsorb anthocyanins, potentially subtracting colour‐active compounds from the must, a phenomenon observed in conventional and hybrid grapes alike.^56^ Colour stability is therefore a critical concern for PIWI wines. The low tannin‐to‐anthocyanin ratio, combined with variable levels of flavonol glycosides, may undermine pigment stabilization over time. In addition, the high content of glycosylated flavonols in cultivars like VLS, JLS, and KHR is consistent with strong expression of Va5GT and Vl3GT and may influence colloidal equilibria and overall phenolic balance. Notably, concentrations of free quercetin above 30 mg L^−1^ are known to cause precipitation in *V. vinifera* wines,^57^ and recent analyses of PIWI wines have reported much higher levels of this compound.^9^ However, recent studies showed that quercetin solubility depends on red wine matrix composition, including anthocyanins, tannins and pH.^58^ The effective solubility threshold for quercetin in PIWI wines may therefore differ from the 30 mg L^−1^ value reported for conventional *V. vinifera* wines. This suggests that standard vinification protocols may not be adequate for PIWI grapes, which require adapted approaches to manage tannin extraction, colour stabilization and flavonol‐related instabilities. Altogether, the metabolic behaviour observed in this PIWI panel reflects a combination of hybrid ancestry and cultivar‐specific enological traits. Although some recurrent tendencies emerged (e.g., often‐high acidity, generally low skin tannins and frequently elevated contents of anthocyanins and glycosylated flavonols), their magnitude and combinations differed markedly among genotypes. These compositional features suggest that conventional winemaking practices may require cultivar‐tailored adjustments, particularly to manage tannin extraction, colour stabilization, and flavonol‐related instabilities. Future multi‐year, multi‐site and multi‐stage ripening studies will be necessary to assess the robustness of these patterns across seasons, environments and developmental stages.

## CONCLUSION

This study provides an integrated molecular and metabolic characterization of five Italian red PIWI cultivars at harvest, highlighting marked cultivar‐specific differences in transcriptional and phenolic profiles. Within this panel, genotype‐dependent modulation of the TOR/SnRK1‐related signalling components and of flavonoid/glycosylation genes was associated with differences in berry composition, particularly in anthocyanins, flavonol glycosides, colour‐related parameters, and extractable tannins. Compared with PN, several (but not all) PIWI cultivars showed higher flavonol glycosides and lower extractable skin tannins, supporting the view that PIWI berries can differ substantially in phenolic balance and potential winemaking behaviour. These results therefore define a first cultivar‐resolved transcriptional‐metabolic framework for Italian red PIWI grapes and support the need for cultivar‐tailored winemaking strategies. Given the limited cultivar panel and the single‐vintage design, with each cultivar analysed at a single harvest stage, broader generalization to PIWI grapes as a group will require validation across additional genotypes, environments, and ripening stages.

## CONFLICT OF INTEREST

The authors declare that they have no conflicts of interest.

## Supporting information


**Table S1.** Identity, breeder‐reported pedigree background, and reported resistance loci of the PIWI cultivars analysed in this study. Breeder's reference codes, pedigree background, and resistance loci/QTL were extracted from VCR 2022.
**Table S2.** List of target genes analysed with the respective ID number, primer sequence, and annealing temperature used in absolute qPCR.
**Table S3.** Least‐squares means (LS means) of transcript levels for sugar‐signalling (TOR3, SnRK1.1, SnRK1.2), flavonoid‐biosynthetic (LAR1, LAR2, ANR, DFR, LDOX, FLS4, FLS5) and glycosyltransferase genes (GAT1, Va5GT, Vl3GT, UFGT, UGT72) in berries of Pinot Noir (PN) and PIWI cultivars. Cultivar abbreviations are as follows: PN, Pinot Noir; VLS, Cabernet Volos; JLS, Julius; EDS, Cabernet Eidos; KNT, Merlot Kanthus; KHR, Merlot Khorus. Values are LS means from the mixed‐model analysis; different letters within a column indicate significant differences among cultivars (*P* < 0.05).

## Data Availability

The data that supports the findings of this study are available in the supplementary material of this article.
